# Supplementary material for: Splice-Junction-Based Mapping of Alternative Isoforms in the Human Proteome
Source: Cell Rep. Author manuscript; Available in PMC 2020 Jan 15. (PMC6961840; doi:10.1016/j.celrep.2019.11.026)
Supplement: 2 [file NIHMS1546469-supplement-2.zip › DF1/PXD006675/Aorta/Aorta_10_MYH11_QCQGQAAQEAAGGGR.pdf]

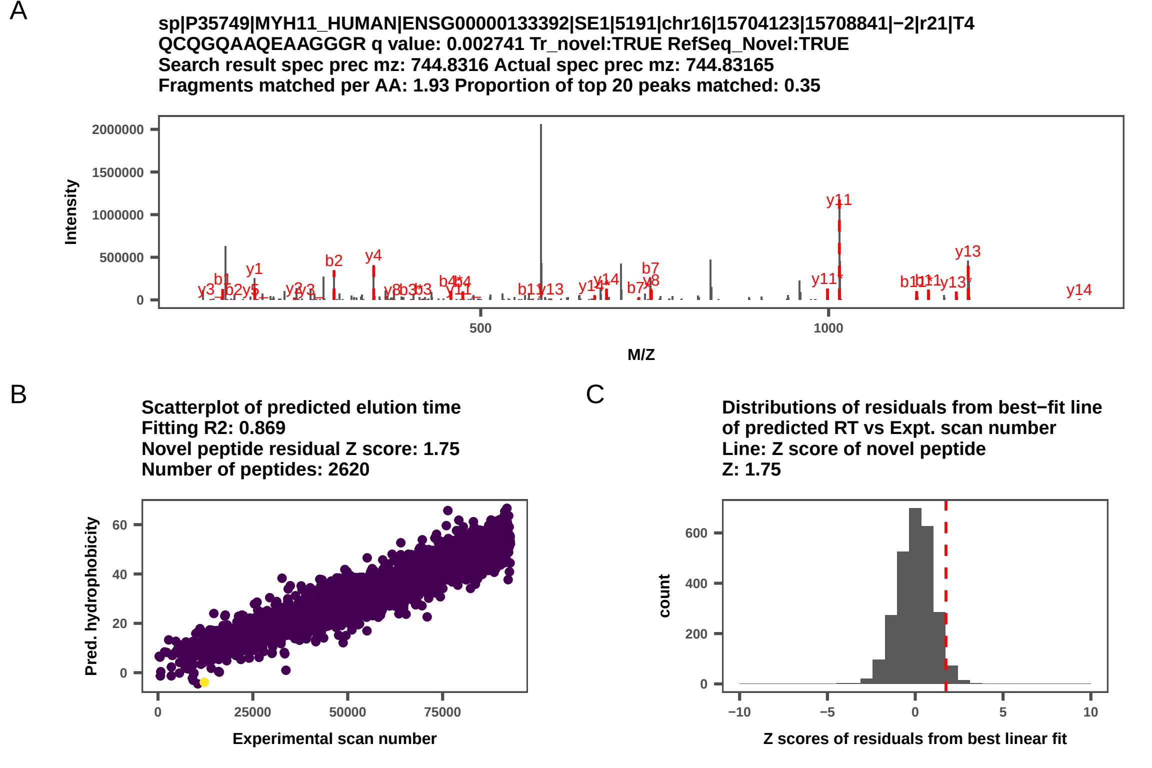

A
sp|P35749|MYH11_HUMAN|ENSG00000133392|SE1|5191|chr16|15704123|15708841|−2|r21|T4
QCQGGQAAQEAAGGGR q value: 0.002741 Tr_novel:TRUE RefSeq_Novel:TRUE
Search result spec prec mz: 744.8316 Actual spec prec mz: 744.83165
Fragments matched per AA: 1.93 Proportion of top 20 peaks matched: 0.35
Intensity
2000000
1500000
1000000
500000
0
y1
b2
y4
b7
y11
y13
y14
500
1000
M/Z
B
Scatterplot of predicted elution time
Fitting R2: 0.869
Novel peptide residual Z score: 1.75
Number of peptides: 2620
Pred. hydrophobicity
60
40
20
0
0
25000
50000
75000
Experimental scan number
C
Distributions of residuals from best−fit line
of predicted RT vs Expt. scan number
Line: Z score of novel peptide
Z: 1.75
count
600
400
200
0
−10
−5
0
5
10
Z scores of residuals from best linear fit
